# Supplementary material for: Graphene Far-Infrared Irradiation Can Effectively Relieve the Blood Pressure Level of Rat Untr-HT in Primary Hypertension
Source: Int J Mol Sci. 2024 Jun 18;25(12):6675. doi: 10.3390/ijms25126675 (PMC11204347; doi:10.3390/ijms25126675)
Supplement: Supplementary file 1 [file ijms-25-06675-s001.zip › ijms-3052935-supplementary.pdf]

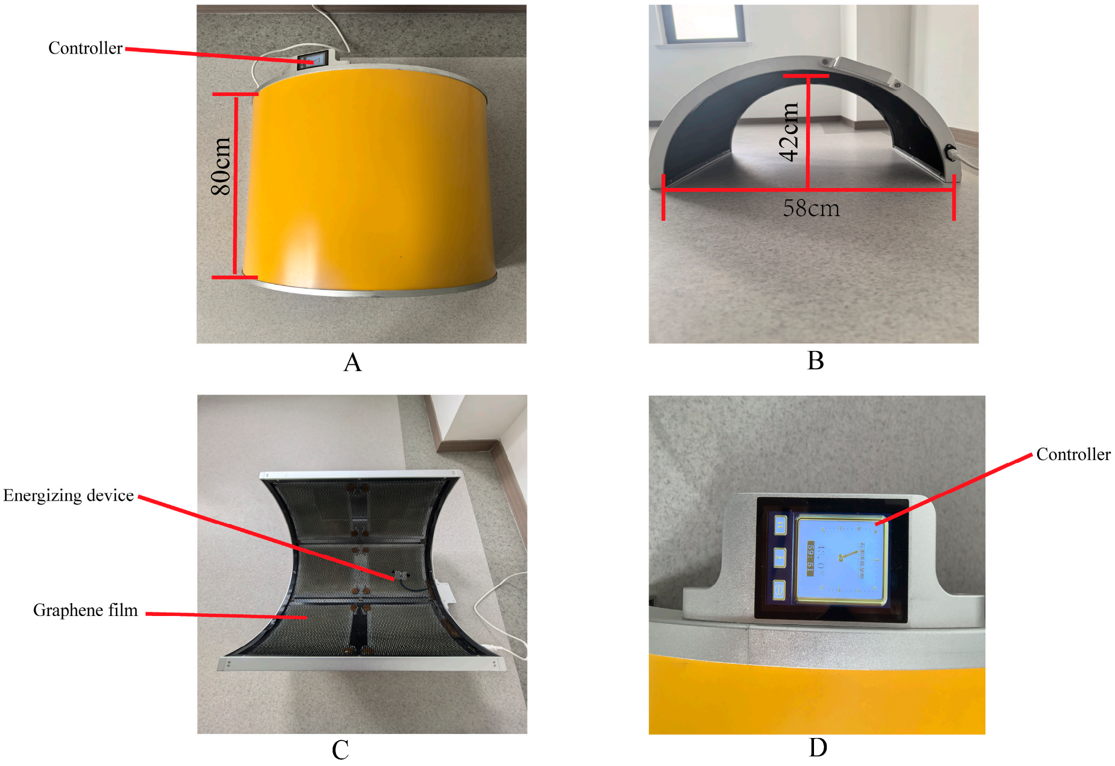

Supplment Figure S1 Graphene far-infrared device. The device is an arch device, the length, width and height are 80cm, 58cm and 42cm respectively, the surface of the device is covered with a graphene single-layer film, and is equipped with an electric heating device, which generates a specific wavelength of infrared ray and a certain amount of heat after being powered on, and the device is equipped with a controller to control the irradiation time and temperature. In the experiment, the squirrel cage was placed directly in the arch device.
